# Genetic Interrelationship Among Newly-Bred Mutant Lines of Wheat Using Diagnostic Simple Sequence Repeat Markers and Phenotypic Traits Under Drought

**DOI:** 10.3390/genes16101210

**Published:** 2025-10-14

**Authors:** Athenkosi Makebe, Hussein Shimelis, Jacob Mashilo

**Affiliations:** 1 African Centre for Crop Improvement (ACCI), University of KwaZulu-Natal, Private Bag X01, Scottsville, Pietermaritzburg 3201, South Africa; makebea@ukzn.ac.za (A.M.); shimelish@ukzn.ac.za (H.S.); 2 Limpopo Department of Agriculture and Rural Development, Towoomba Research Centre, Bela-Bela 0480, South Africa

**Keywords:** bread wheat, genetic diversity, mutagenesis phenotyping, SSR marker

## Abstract

Background/Objectives: Induced mutagenesis is vital in genetic enhancement and trait discovery, for genetic analysis and breeding of novel crop varieties with desirable product profiles. Understanding the genetic relationships among newly developed mutant genotypes enables targeted selection and genetic recombination. Therefore, the objective of the current study was to assess the genetic diversity among mutant bread wheat genotypes developed through ethyl methanesulfonate (EMS) mutagenesis using phenotypic traits and diagnostic simple sequence repeat (SSR) markers to identify novel mutants and traits for breeding. Methods: Sixteen advanced (M6) mutant lines, one parental genotype, and three check varieties were genetically profiled using ten diagnostic SSR markers. The genotypes were evaluated for agronomic traits under drought-stressed (DS) and non-stressed (NS) conditions using a 10 × 2 alpha lattice design with two replications. Results: The SSR markers revealed a total of 21 alleles, with an average of 2.10 alleles per locus. An average polymorphic information content (PIC) of 0.51 was computed, revealing moderate informativeness of the genetic markers. Significant (*p* < 0.05) differences were observed among the test genotypes for key agronomic traits under NS and DS conditions. Grain yield positively and significantly (*p* < 0.001) correlated with plant height (r = 0.79), number of productive tillers (r = 0.82), root biomass (r = 0.77), shoot biomass (r = 0.74), spike length (r = 0.74), total biomass (r = 0.74), and thousand-seed weight (r = 0.64), under DS conditions. Principal component analysis explained 78.03 and 87.14% genotype variation for assessed agronomic traits under DS and NS conditions, with total biomass, shoot biomass, root biomass, productive tiller, plant height and grain yield as key traits contributing the most variation in the test genotypes. Conclusions: Wheat mutants LMA16, LMA44, and LMA53 were identified as genetically distinct and high yielders under drought stress conditions and recommended for production in rain-fed environments. The selected mutants are a valuable source of genes for wheat improvement programs.

## 1. Introduction

Common wheat (*Triticum aestivum* L., 2n = 6× = 42, AABBDD) is a vital commodity crop contributing to food and nutrition security and economies globally. With an annual production of more than 797 million tons, wheat contributes approximately 21% of the global food supply [1]. It serves as a major source of protein and energy to approximately 4 billion people [2]. Wheat grains are rich in proteins (i.e., glutenin and gliadin), carbohydrates, vitamins (i.e., riboflavin, thiamine, and niacin), essential minerals (i.e., Cu, Mg, Zn, Fe, and P), and fiber [3,4]. Other key and notable nutritional profiles of wheat include high contents of phenolic acids, flavonoids, anthocyanins, and carotenoids [5,6]. Thus, wheat is a vital source of essential nutrients and bioactive compounds for human well-being.

Global food demand is increasing rapidly and is projected to double by 2050 due to population growth and urbanization [7,8]. To meet the demand, wheat production must increase by more than 60% by the year 2050 to feed more than 9 billion people [9,10]. It is forecasted that climate change, which manifests in various forms including prolonged periods of drought, extreme temperatures, and flooding, will intensify and cause significant yield losses in wheat [11,12]. Drought stress is a major climate-change-driven environmental constraint affecting sustainable wheat production and productivity worldwide, with yield losses varying between 50 and 90% [13]. Climate change will pose significant challenges for wheat production, especially under rainfed production systems.

Genetic gains for grain yield in wheat are reported to be slower under drought-prone environments compared to irrigated conditions [14]. For example, genetic gains of 40 kg ha^−1^ yr^−1^ were reported under rainfed and drought-prone conditions compared to high yield gains of 160 kg ha^−1^ yr^−1^ under irrigated conditions [15]. Joudi, et al. [16] reported genetic yield gains of 20 kg ha^−1^ yr^−1^ and 31 kg ha^−1^ yr^−1^ in wheat under dry-land and irrigated conditions, respectively. It is estimated that agricultural land surface temperature will increase by 0.06 °C annually, accompanied by a reduction in rainfall by 16.0 mm per annum [17]. The decrease in rainfall and increase in temperature threaten rainfed wheat production [18]. The projected decline in grain yield due to climate change poses a significant threat to food security and the long-term sustainability of wheat cultivation. There is an urgency for the development of innovative solutions to enhance wheat production, resilience, and sustainability.

Breeding of wheat varieties that combine high grain yield and stability under drought stress conditions is crucial to boost yield gains to ensure food security and enhance climate resilience in wheat production systems. To develop high-yielding and climate-smart wheat varieties, mutagenesis has played a key role in generating new genetic stocks for the improvement of economic traits, including grain yield and quality, phenological traits and disease resistance, and heat and drought tolerance [19,20]. Chemical mutagenesis using ethyl methanesulfonate (EMS) is a widely used approach for breeding novel wheat mutants. For example, Komura, et al. [21] used EMS to create early-flowering wheat mutants, while a short-stature wheat mutant line with enhanced grain yield was developed by [22]. Recently, Kumar, et al. [23] developed an EMS-mutagenized wheat population with improved agronomic traits (i.e., grain yield, tiller number, and peduncle length) and increased micronutrient content (i.e., Ca, Cu, Fe, and Zn). Furthermore, EMS mutagenesis aided the development of wheat mutant lines resistant to both Fusarium crown rot and head blight [24]. In South Africa, OlaOlorun, et al. [25] developed a wheat population with enhanced root biomass allocation, drought tolerance, and improved yield potential. Overall, these findings validate the potential of EMS mutagenesis in the development of novel wheat varieties to enhance genetic gains for key traits, which is crucial to safeguarding food security.

Understanding the genetic diversity in wheat genetic resources, including mutant genotypes, is crucial for germplasm preservation and identification of diverse parental lines. Phenotypic analysis based on morphological traits has been used for germplasm characterization in wheat [26,27]. Molecular-marker-based genetic analysis complements traditional phenotyping of crop genetic resources independent of environmental factors compared to classical phenotyping. Among the various molecular marker systems, SSR markers have been widely used in genetic diversity studies, genome mapping, QTL mapping, and marker-assisted selection, including in drought tolerance breeding programs [28,29]. SSR markers are highly polymorphic, co-dominant, chromosome-specific, and are spread across the genome. SSR-based genetic analysis enables the identification of key genes linked to various agronomic traits in wheat [30]. For example, Khan et al. [31] identified marker Xbarc49 with association with stay-green under heat stress conditions. Three SSR markers, gwm165, gwm325, and gwm539, were reported to be linked with plant height in wheat [32].

Wheat is a key commodity crop in South Africa, which is extensively produced under rainfed conditions [33]. For example, production of winter/intermediate wheat production occurs mainly under dryland conditions of the Free State province. Dryland spring wheat is mainly produced in the Western Cape province, characterised by hot and dry summers [34,35]. South Africa is a water-scarce country, and climate modelling using decision support system for agrotechnology transfer (DSSAT) indicated a high probability of declining wheat production in major production areas due to climate-change-driven declines in rainfall and recurring droughts [36]. Nxumalo, et al. [37] used the Standardized Precipitation Index (SPI) to project increased drought frequencies and reported significant yield losses in wheat across South Africa, with the Western Cape and Northern Cape being particularly vulnerable. To mitigate the effects of climate change on wheat cultivation, especially in dryland production areas of South Africa, the African Centre for Crop Improvement initiated a breeding program to develop new generation wheat varieties that are drought-tolerant and high-yielding. Mutation breeding using EMS was applied to the highly drought and heat-tolerant wheat cultivar LM43 sourced from the International Maize and Wheat Improvement Centre (CIMMYT) [25,38]. This aided the development of 16 novel mutants with desirable agronomic traits, including high yield performance, drought tolerance and enhanced root and shoot biomass allocation. The novel mutants are valuable genetic resources for the selection of novel mutants and traits to introgress genes to widely cultivated varieties for abiotic stress tolerance and the development of locally adapted varieties. Therefore, the objective of the current study was to assess the genetic diversity among mutant bread wheat genotypes developed through ethyl methanesulfonate (EMS) mutagenesis using phenotypic traits and diagnostic simple sequence repeat (SSR) markers to identify novel mutants and traits for breeding.

## 2. Materials and Methods

### 2.1. Plant Materials

The present study evaluated 20 bread wheat genotypes, comprising 16 advanced mutants (M6) selected with superior agronomic performance and root biomass allocation under drought stress and non-stress conditions. The other four genotypes include the mutant parental genotype sourced from the International Maize and Wheat Improvement Centre (CIMMYT) and three varieties. The names and pedigree of the genotypes are presented in Table 1.

### 2.2. Genotyping

#### Selection of SSR Markers and Genotyping

To assess the genetic diversity of the 20 wheat genotypes, ten diagnostic SSR markers were used (Table 2). The markers were selected based on their informativeness and ability to discern wheat genotypes under heat and terminal drought tolerance [39,40,41]. Seeds of the selected genotypes were grown in seedling trays until the 4-leaf stage. DNA of leaf samples was extracted from two-week-old seedlings using the modified cetyltrimethylammonium bromide (CTAB) protocol. Leaf tissues were placed into microcentrifuge tubes and mixed with 500 μL of CTAB buffer, and it was incubated for 30 min in a hot water bath at 70 °C. Tubes were centrifuged at 3500 rpm for 10 min. After the centrifugation, the supernatant was collected and transferred into a microcentrifuge tube, then chloroform: isoamyl alcohol (24:1) was added to the microcentrifuge tube and mixed gently. Then the DNA was precipitated and separated from the solution by adding ethanol. The DNA pellet was dried and resuspended in a solution of TE buffer to stabilize and preserve the DNA. The quality of the DNA was estimated using a Nano-drop^®^ ND-1000 spectrophotometer (Nanodrop Products, Wilmington, DE, USA).

### 2.3. PCR Amplification

Polymerase chain reaction (PCR) was performed using 12 µL of reaction mixture containing 1 × PCR buffer 2.5 mm Mg^2+^, 0.2 µL each of dinucleotidetriphosphates. The PCR began with initial denaturation at 94 °C for 2 min, followed by 33 cycles of denaturation at 94 °C for 30 s each. Further, PRC annealing was performed at 63 °C for 30 s, followed by extension at 72 °C for 45 s, with a final extension step at 72 °C lasting 20 min. The resultant products were labelled using a fluorescent marker and separated with capillary electrophoresis using an ABI 3130 automatic sequencer (Applied Bioscience, Foster City, CA, USA), and GeneMapper 4.1 was used for fragment analysis.

### 2.4. Phenotyping Protocols

Two separate experiments were conducted under drought-stress (DS) and non-stress (NS) conditions in a controlled glasshouse and field environments. The glasshouse experiment was conducted at the Controlled Environment Facility (CEF) of the University of KwaZulu-Natal (29.6213° S, 30.3966° E). The average day/night temperatures in the glasshouse were maintained at 25 °C/15 °C, whereas the relative humidity was 45% and 60% during the study, respectively. The greenhouse experiment was laid out in a 10 × 2 alpha lattice design with two replications. Ten seeds were planted in 5 L capacity plastic pots using composted pine bark growth media, and they were thinned to 5 plants per pot 3 weeks after emergence. Automated drip irrigation was used to supply water and fertilizer directly to the pots. The fertilizer application was integrated into a computerized drip irrigation system, with a rate of 300 kg N ha^−1^ and 200 kg P2O5 ha^−1^. DS was imposed at 50% flowering until physiological maturity by withholding irrigation until the soil water content reached 30% field capacity. Plants were watered regularly for NS conditions to maintain soil moisture close to field capacity. A tensiometer (GTDSMM500, General Tools and Instruments, Secaucus, NJ, USA) was used to measure soil moisture levels in the pot during the experiment.

The field experiment was carried out at Ukulinga Research Farm (29°40′ S, 30°24′ E) of the University of KwaZulu-Natal, Pietermaritzburg, South Africa. The field experiment was laid out as a 10 × 2 alpha lattice design with two replications. The genotypes were grown in a specially designed plastic mulch rainout with an underground drip irrigation system. Each plot was a 1.5 m long single row per genotype, where two seeds were planted per planting station at 10 cm in row spacing and 45 cm between rows. Drought stress was induced at 50% heading by reducing irrigation to 35% capacity, while full irrigation was maintained for the NS conditions. Soil moisture content in the field was measured using a digital moisture sensor (HOBO UX120, Onset, Bourne, MA, USA). Basal fertilizer consisting of nitrogen (N), phosphorus (P), and potassium (K) was applied at a rate of 120:30:30 kg ha^−1^ (N:P:K) at planting based on the recommendation of wheat production in South Africa [42]. Weeds were removed manually, while aphids and other insect pest infestations were controlled using insecticides.

### 2.5. Agronomic Data Collection

Agronomic data were collected for the following traits: days to 50% heading (DTH) calculated as the number of days from sowing to the date when half of the plants in a pot or plot had fully developed spikes. Days to 90% maturity (DTM) was calculated as the number of days from sowing to the date when 90% of the plants senesced. Plant height (PH, expressed in cm) was measured from ground level to the top of the spike, excluding the awns. The number of productive tillers (PTN) was recorded at physiological maturity, while shoot biomass (SB, expressed in grams) was measured by weighing the above-ground plant material. After carefully washing the roots, root biomass (RB, g) was measured as the weight of the below-ground plant parts. SB and RB were oven dried for 72 h at 65 °C. Spike length (SL, cm) was measured from the base of the spike to the tip, excluding awns. Two hundred seeds were counted from each genotype, weighed in grams using a digital balance, and multiplied by 5 to obtain the thousand kernel weight (TKW). Grain yield was measured as the mean weight (grams) of grains harvested from a plot. The field data from the field were extrapolated based on five plants to be consistent with the greenhouse data.

### 2.6. Data Analysis

#### 2.6.1. Agronomic Data Analysis

The agronomic data were subjected to analysis of variance (ANOVA) using GenStat 18th Edition (VSN International, Hempstead, UK). Differences between genotype means were assessed for statistical significance using Fisher’s Least Significant Difference (LSD) test at the 5% significance level. Pearson correlation coefficients were constructed to measure interrelationships among the traits using the corrplot procedure [43] in R version 4.2.0 [44]. Principal component analysis (PCA) was performed with R based on the correlation matrix for both NS and DS conditions, to identify influential traits. PCA biplots were generated for the NS and DS conditions across the testing environments using the factoextra package in R software version 4.3.3 [45]. 

#### 2.6.2. Marker Data Analysis

Genetic diversity analyses were performed using GenAlex version 6.5 [46]. The following genetic parameters were calculated: the total number of alleles per locus (Na), the number of effective alleles per locus (Ne), observed heterozygosity (Ho), expected heterozygosity (He), and fixation index (FIS). PIC calculated using the formula PIC = 1 − ⅀Pij2, where Pij is the frequency of the jth allele of the ith locus [47]. A dendrogram was constructed from the distance matrix using the neighbour-joining method in the Darwin 6.0.5 software [48]. A tanglegram was used to compare phenotypic and genotypic hierarchical clusters using the “dendextend package” [49] in R software version 4.3.3 [44].

## 3. Results

### 3.1. Genetic Diversity Using SSR Markers

#### 3.1.1. Genetic Parameters

Table 3 presents genetic diversity parameters generated using the SSR markers. WMC78 was a monomorphic marker, while the rest were polymorphic. The number of alleles (Na) varied from 1 (WMC78) to 2.67 (Xgwm484, XWMC596, and WMC179) with an average of 1.97. The number of effective alleles (Ne) ranged from 1 (WMC78) to 2.53 (WMC179) with a mean of 1.82 per locus. Shannon’s information index (I) ranged from 0 to 0.90 with an average of 0.54 across all the markers (Table 3). The observed heterozygosity ranged from 0 (markers GWM337, WMC532, and WMC78) to 1.00 (Xgwm132) with a mean of 0.42, while the expected heterozygosity ranged from 0 (WMC78) to 0.57 (WMC179), with an average of 0.35. The fixation index (F) varied from −1 to 1 with an average of 0.24. PIC values varied from 0.00 (WMC78) to 0.83 (XWMC596) with an average of 0.51. The overall results suggest that the SSR markers used in this study were both reliable and useful for genetic analysis.

#### 3.1.2. Principal Coordinate Analysis (PCoA)

**Figure 1:**
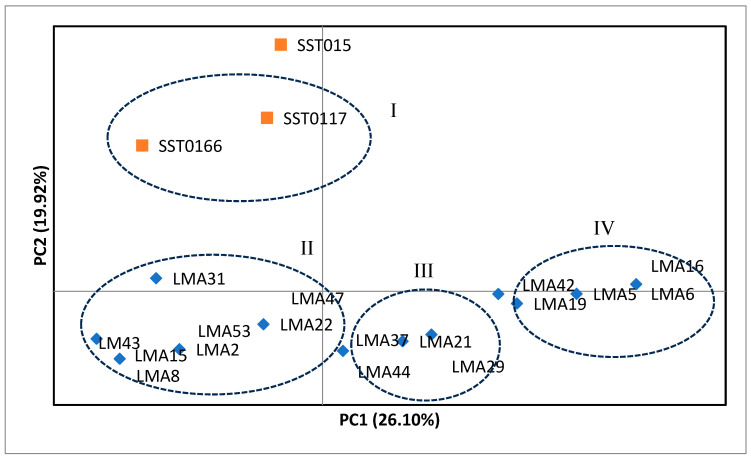
Principal coordinate analysis (PCoA) showing genetic relationships of check wheat varieties and mutant lines based on 10 simple sequence repeat markers.

Principal coordinate analysis showing genetic relationships among the wheat mutant lines and standard varieties is shown in Figure 1. Principal components 1 (PC1) and 2 (PC2) accounted for 26.10% and 19.92% of the total variation, respectively. There were four (denoted as I, II, III, and IV) distinct genetic groups based on PcoA (Figure 1). The check varieties SST015, SSTO117, and SSTO166, were grouped on the top left quadrant and distinctly separated from the other test genotypes. The mutant lines were grouped into three distinct genetic groups. PCoA did not group the mutants based on their drought tolerance levels, and mutants with different tolerance categories were allocated across the coordinate axes. For example, drought-tolerant mutants such as LMA53 and LMA2 were grouped (i.e., group II) with moderately tolerant mutants such as LMA15 and LMA31. Group III consisted of four mutant lines, while group IV consisted of five mutant lines.

#### 3.1.3. Hierarchical Cluster Analysis

**Figure 2:**
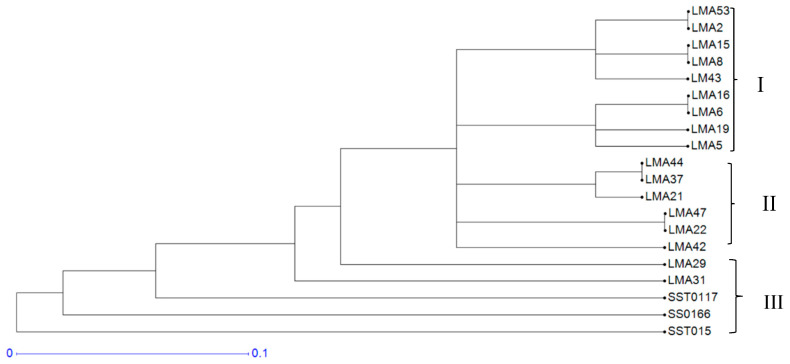
Dendrogram showing genetic diversity among the 20 wheat genotypes, which included check varieties and mutant lines, using 10 polymorphic Simple Sequence Repeat markers.

Genetic interrelationships among the 20 wheat genotypes were further assessed using a dendrogram constructed from a genetic distance dissimilarity matrix (Figure 2). The dendrogram grouped the genotypes into three clusters. Clusters I and II comprised the mutant lines, including the parental genotype. Cluster III comprised the check varieties SST0117, SS0116, and SST015.

### 3.2. Phenotyping Based on Agro-Morphological Traits

#### 3.2.1. Genotypic Variation for Agronomic Performance

Combined analysis of variance revealed significant (*p* < 0.001) genotypic effects for all traits except RSR, while water regime was significant (*p* < 0.05) for all recorded traits except DTH and SL (Table 4). Environmental effects were significant (*p* < 0.001) for recorded traits. The genotype × water regime interaction effect was significant (*p* < 0.05) for DTH, TB, and TSW, while the genotype × environment interaction effect exerted a significant (*p* < 0.05) effect on all traits except DTM. The interaction between genotype, environment, and water regime was significant (*p* < 0.05) for DTM, TB, TSW, and GY.

#### 3.2.2. Agronomic Performance Under Non-Stressed and Drought-Stressed Conditions

**Figure 3:**
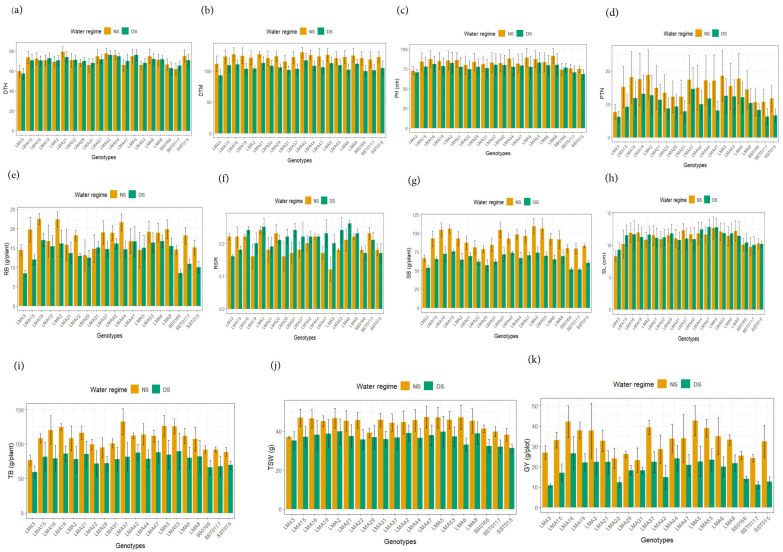
Mean values for agronomic traits of 20 wheat genotypes evaluated under drought-stressed (DS) and non-stressed (NS) conditions across glasshouse and field conditions. DTH: days to 50% heading, DTM: days to 90% maturity, PH: plant height, PTN: productive tiller number, RB: root biomass, RSR: root-shoot ratio, SB: shoot biomass, SL: spike length, TB: total biomass, TSW: thousand seed weight, GY: grain yield. DS, drought stress, NS, non-stressed. (**a**) days to 50% heading; (**b**) days to 90% maturity; (**c**) Plant height; (**d**) productive tiller number; (**e**) root biomass; (**f**) root-shoot ratio; (**g**) Shoot biomass; (**h**) Spike length; (**i**) Total biomass; (**j**) Thousand seed weight; (**k**) Grain yield.

Figure 3 presented the combined mean values of the studied agronomic traits among the 20 wheat genotypes evaluated under DS and NS conditions in the glasshouse and field environments. The mean DTH under DS and NS were 70 and 71 days, respectively (Figure 3a). LM43 was early flowering at 57 days, while LM5A and LMA42 were late flowering at 76 days under DS conditions. Under NS conditions, LM43 was early flowering genotype (60 days), followed by SST0117 (62 days), whereas LMA4 and LMA21 were late flowering genotypes at 78 and 79 days, respectively. For DTM, LM43 and SS0166 were early maturing (<100 days), while LMA42 was late maturing at 117 days under DS conditions. Under NS conditions, LM43 was early maturing (112 days), compared to the late-flowering genotypes LMA42, LMA16, and LMA21 (>125 days) (Figure 3b). The mean PH under DS condition was 77.40 cm, with LMA53, LMA2, and LMA16 being the tallest genotypes at 82.91, 82.59 and 81.33 cm and SST015 the shortest genotype (68.18 cm) (Figure 3c). Under NS conditions, the average PH was 82.79 cm with LMA8, LMA5, and LMA44 being the tallest genotypes at 91.37, 89.16, and 88.06 cm and the genotypes SST015, SS0166, and LM43 being the shortest genotypes at 74.79, 74.05, and 72.74 cm, in that sequence.

Under DS conditions, genotypes LMA19 and LMA37 produced high PTN (>13), while LM43, SST0117, and SST015 recorded fewer PTN (<7) (Figure 3d). Under NS condition, LMA16, LMA5 and LMA2 produced high PTN (>17), whereas LM43, SS0166, SST0117 and SST015 produced few PTN (<12). The mean RB under DS condition was 13.92 g, where test genotypes LMA42, LMA2, LMA53, LMA47, LMA6 and LMA16 recorded the highest RB (>16 g), compared to low RB (<9 g) recorded for LM43 and SS0166 (Figure 3e). Under DS condition, LMA19, LMA5 and LMA42 produced the highest SB (>73 g/plant), while LMA29, LM43, SS0166 and SST0117 recorded lower SB (<60 g/plant) (Figure 3g). Under NS condition, genotypes LMA37, LMA16, LMA19, LMA53 and LMA5 recorded higher SB (>100 g/plant), compared to low SB (<80 g/plant) recorded for LM43, LMA29, SST0117 and SS0166. Under DS conditions, LMA44, LMA5, and LMA47 recorded the longest SL (>12 cm), compared to the shortest SL exhibited by LM43 at 9.27 cm. Under NS condition, LMA53, LMA8, LMA37, and LMA5 recorded the longest SL (>12 cm), compared to the shortest SL (<10 cm) recorded for LM43 and SST0117 (Figure 3h). Under DS condition, TB was highest (>87 g/plant) for LMA53, LMA47 and LMA42, while SST015, SST0117, SS0166 and LM43 recorded low TB (<70 g/plant). LMA37 produced the highest TB (>132 g/plant), while SST015 and LM43 recorded low TB (<90 g/plant) under NS conditions. Under DS conditions, LMA2, LMA5 and LMA42 recorded high TSW (>39 g), while SST015 produced lower TSW (<32 g) (Figure 3j). Genotypes LMA47, LMA6, and LMA5 recorded highest TSW (>47 g), while SST0117, LMA29, SST015, and LM43 recorded low TSW (<40 g). Genotypes LMA16, LMA44, and LMA53 recorded the highest GY (>23 g/plot), while SST015, LMA22, SST0117, and LM43 recorded the lowest GY (<13 g/plot) under DS condition (Figure 3k). Genotypes LMA5 and LMA16 recorded the highest GY (40 g/plot) under NS conditions, whereas SST0117, LMA22, and LMA31 recorded low GY (25 g/plot).

#### 3.2.3. Associations Among Phenotypic Traits Under Contrasting Water Regimes

**Figure 4:**
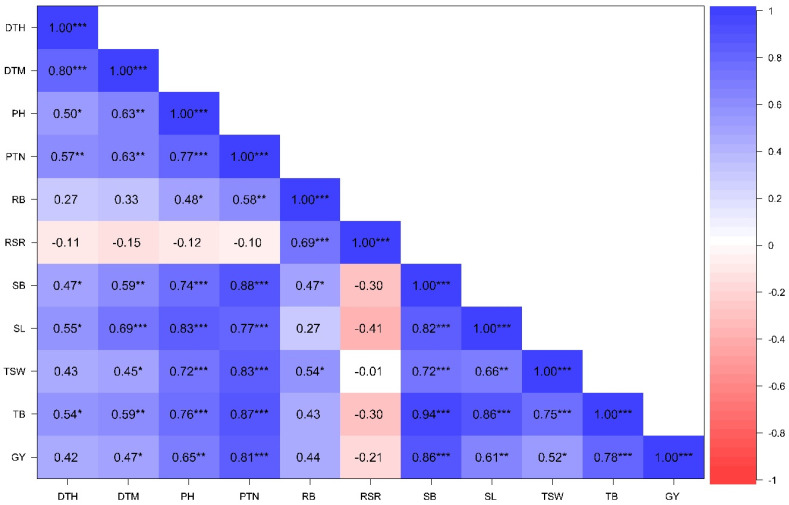
Correlation coefficient of phenotypic traits of 20 wheat genotypes evaluated under non-stressed conditions across glasshouse and field environments. DTH: days to 50% heading; DTM: days to 90% maturity; PH: plant height; PTN: productive tiller number; SB: shoot biomass; RB: root biomass; TB: total biomass; RSR: root–shoot ratio; SL: spike length; TSW: thousand-seed weight; GY: grain yield; *: significant at *p* < 0.05; **: *p* < 0.01; ***: *p* < 0.001.

**Figure 5:**
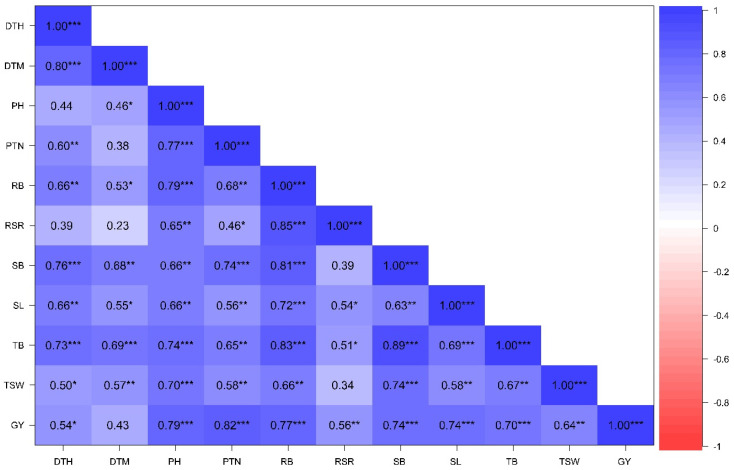
Correlation coefficient of phenotypic traits of 20 wheat genotypes evaluated under drought-stressed conditions across glasshouse and field environments. DTH: days to 50% heading; DTM: days to 90% maturity; PH: plant height; PTN: productive tiller number; SB: shoot biomass; RB: root biomass; TB: total biomass; RSR: root–shoot ratio; SL: spike length; TSW: thousand-seed weight; GY: grain yield; *: significant at *p* < 0.05; **: *p* < 0.01; ***: *p* < 0.001.

Pearson correlation analysis showing trait association under NS and DS conditions across glasshouse and field environments is presented in Figure 4 and Figure 5. Positive and significant associations were recorded between GY and PH (r = 0.79; *p* ≤ 0.001), PTN (r = 0.88; *p* ≤ 0.001), RB (r = 0.77; *p* ≤ 0.001), SB (r = 0.74; *p* ≤ 0.001), SL (r = 0.74; *p* ≤ 0.001), TB (r = 0.70; *p* ≤ 0.001), and TSW (r = 0.64; *p* ≤ 0.001) under DS condition. Positive and significant associations (r > 0.70; *p* ≤ 0.001) were recorded between RB with GY, TB, SL, SB, and RSR under DS conditions. Furthermore, RB showed moderate and positive correlations with PH, PTN, RSR, SB, and TSW under NS conditions but a non-significant association with GY. Trait associations were stronger under DS compared to NS conditions.

#### 3.2.4. Multivariate Relationships

PCA showing the contribution scores and cumulative variations for assessed phenotypic traits under DS and NS conditions is presented in Table 5. Under DS conditions, the first two principal components (PCs) accounted for 78.03% of the total variation. PC1 was positively associated with GY, PH, PTN, RB, PTN, TSW, SB, SL, and TB. The second PC, which contributed 10.68% of the total variation, was positively associated with RSR. However, DTH and DTM contributed negatively. Under NS conditions, the first three PCs contributed 87.14% of the total variation. The first PC explained 61.97% of the total variance, and the major positive contributing traits were GY, PH, PTN, SL, SB, TB, and TSW. RB and RSR contributed negatively to PC2, which accounted for 16.11% of the total variation. PC3 accounted for 9.06% of the total variation, where DTH and DTM contributed negatively.

#### 3.2.5. Principal Component Biplots

**Figure 6:**
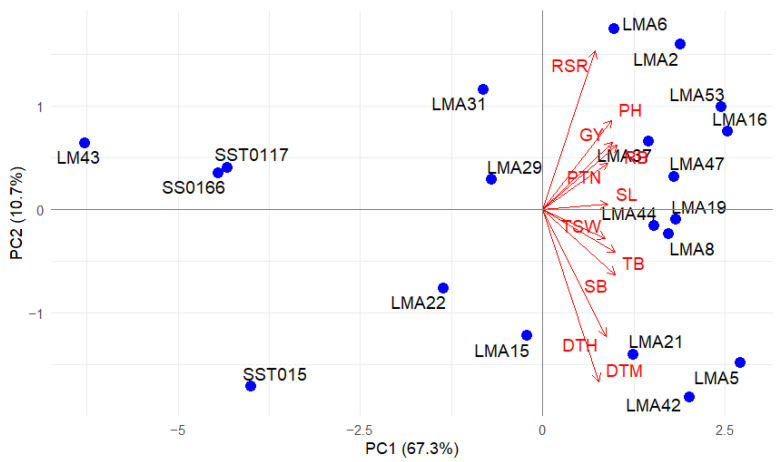
Principal component biplot depicts the grouping of 20 bread wheat genotypes evaluated under DS conditions. DTH, days to 50% heading; DTM, days to 90% maturity; PH, plant height; PTN, productive tiller number; SB, shoot biomass; RB, root biomass; TB, total biomass; RSR, root–shoot ratio; SL, spike length; TSW, thousand-seed weight; GY, grain yield.

**Figure 7:**
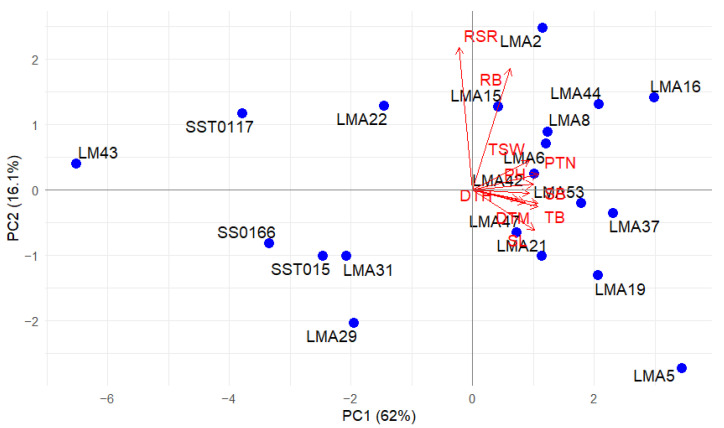
Principal component biplot depicts grouping of 20 bread wheat genotypes evaluated under NS conditions. DTH, days to 50% heading; DTM, days to 90% maturity; PH, plant height; PTN, productive tiller number; SB, shoot biomass; RB, root biomass; TB, total biomass; RSR, root–shoot ratio; SL, spike length; TSW, thousand-seed weight; GY, grain yield.

The association among the traits and wheat genotypes under DS and NS conditions across glasshouse and field environments was visualized in a principal component biplot (Figure 6 and Figure 7). Narrow angles (<45°) between dimension vectors indicate significant correlation of traits, while the most influential traits to differentiate the genotypes have the longest vector lines. Under the DS condition, all the traits contributed positively to PC1. Narrow angles were observed between GY, PH, RB, PTN, and RSR under DS conditions. Mutant lines LMA16, LMA37, and LMA53 were plotted closer the vector for GY. Under NS conditions, all the traits contributed positively to PC1 except for RSR. LMA53 and LMA37 were plotted closer to vectors of GY, TB, and SB, whereas genotypes LMA16, LMA44, LMA2, and LMA8 were grouped based on high RB.

#### 3.2.6. Cluster Analysis Based on Phenotypic Traits

**Figure 8:**
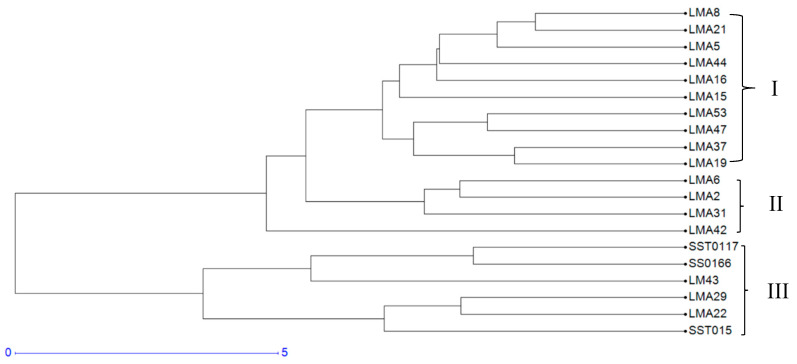
Hierarchical clustering of 20 wheat genotypes based on phenotypic traits measured under drought stress (DS) conditions.

**Figure 9:**
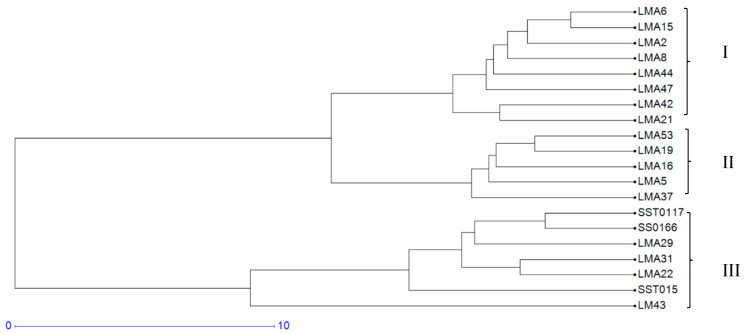
Hierarchical clustering of 20 wheat genotypes based on phenotypic traits measured under NS conditions.

Figure 8 reveals the clustering of the 20 wheat genotypes for agronomic traits into three distinct clusters under the DS condition. Cluster I consisted of ten genotypes, followed by Cluster III, which consisted of six genotypes, and Cluster II, which consisted of four genotypes. Cluster II genotypes had higher RSR, while Cluster III comprised genotypes with low GY, early flowering, and lower RB. Similarly, under the NS condition, the hierarchical clustering grouped the genotypes into three different clusters (Figure 9). Clusters I, II, and III consisted of eight, seven, and five genotypes, respectively. Cluster I contained genotypes such as LMA2, LMA44, LMA8, and LMA15, which exhibited high RB. Cluster II contained genotypes with higher GY, TB, and SB; cluster III comprised genotypes with low GY and TSW.

#### 3.2.7. Phenotypic and Genotypic Hierarchical Cluster

**Figure 10:**
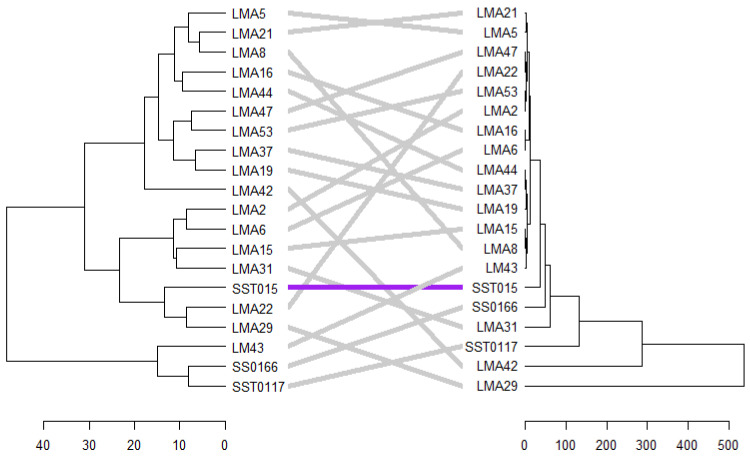
Tanglegram comparison of phenotypic and genotypic hierarchical clusters of 20 wheat genotypes based on 10 SSR markers and phenotypic traits measured under drought stress conditions.

**Figure 11:**
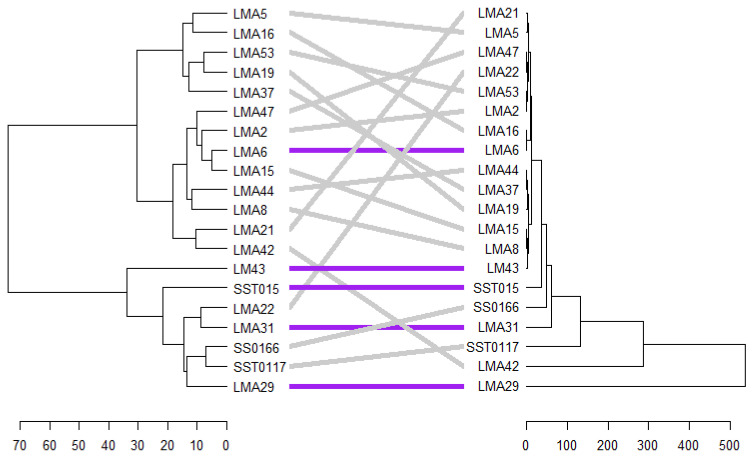
Tanglegram comparison of phenotypic and genotypic hierarchical clusters of 20 wheat genotypes based on 10 SSR markers and phenotypic traits measured under non-stress conditions.

A tanglegram comparing clustering patterns of the test genotypes based on phenotypic traits and SSR markers under DS conditions is shown in Figure 10. An entanglement score of 0.25 was recorded, indicating low dissimilarity between genetic and phenotypic clustering (Figure 9). SST015 is the only genotype that maintained its position and cluster under drought stress conditions. Under NS conditions, an entanglement score of 0.25 was recorded. Five genotypes (i.e., LMA6, LM43, SST015, LMA31, and LMA29) showed similar match patterns between the two tanglegrams (Figure 11).

## 4. Discussion

The SSR markers used in the current study detected low allele number (i.e., 21 alleles in total) and alleles per locus (i.e., 2), an indication of low genetic diversity among the mutant lines. Further, low allele number per locus is an indication of ineffective EMS mutagenesis, probably resulting in low mutation rates. Additionally, the low genetic diversity could also be attributed to a common genetic background, especially provided the mutants were derived from LM43 (ROLF07*2/6/PVN//CAR422/ANA/5/BOW/CROW//BUC/PVN/3/YR/4/TRAP#1), a heat and drought tolerant wheat variety developed by CIMMYT and high selection pressure imposed in early generations for desirable agronomic traits. Nonetheless, the mean number of alleles per locus reported in the current study is similar to previous findings by Verma, et al. [50] and Meena, et al. [51] when assaying 81 and 36 wheat genotypes. Contrastingly, Mahmood, et al. [52] and Şen and Sarsu [53] reported a high number of alleles per locus among wheat mutants. The contrasting reports are attributed to differences in optimum dosage, duration, treatment condition, and the number of SSR markers used in each study. Polymorphism information content (PIC) provides insight into allele presence and distribution [54], serving as a measure of marker informativeness. In the present study, the average PIC value was 0.51, indicating that the SSR markers used are reliable and recommendable for genetic analysis of commercial and mutant wheat genotypes. For example, the SSR loci XWMC596, WMC179, and Xgwm484 were the most informative and highly discriminating of the test genotypes (Table 3). These markers have been previously reported to be highly discriminating of wheat genetic resources [27, 55]. Contrastingly, the SSR loci GWM337 and WMC78 were the least informative as detected in the present study. This contrasts with earlier findings by Mkhabela, et al. [56], where these primers exhibited high PIC values among wheat germplasm. The contrast could be attributed to differences in the genetic architecture of the wheat germplasm. 

Shannon’s information index is an important measure of genetic diversity within a population. In the current study, the average Shannon’s information index of 0.64 indicated moderate levels of genetic diversity among the test genotypes. Tahir, et al. [57] reported Shannon’s Information index of 0.58 among 20 wheat genotypes using eight SSR markers, agreeing with the present findings. The mean observed heterozygosity value of 0.42 reported in the current study is higher than the values of 0.32 and 0.23 reported by Demirel and Demirel [58] and Urquijo-Zamora, et al. [59] in wheat. The mean heterozygosity of 0.35 also confirmed moderate genetic diversity of the assayed genotypes, and is relatively lower than values of 0.55 and 0.46 reported by Farhangian-Kashani, et al. [54] and Dagnaw, et al. [60] when assessing genetic diversity in wheat using SSR markers. The low genetic diversity of the tested mutants may hinder crop improvement initiatives due to limited allelic variation, which is necessary for effective selection. Despite the low allele frequency and moderate gene diversity, the present study revealed genetically differentiated mutants based on the Hierarchical cluster analysis (Figure 2). The clustering was based on genetic descent and common ancestry, such that commercial-grown wheat varieties SST0117, SS0116, and SST015, and mutant genotypes were grouped distinctly. The mutants LMA6, LMA42, LMA53, LMA29, LMA16, LMA37 and LM43 were genetically differentiated and differentially isolated among the mutants in clusters I and III. Crosses derived from these unique mutants can develop a novel wheat population and increase genetic gains for agronomic traits under dryland and irrigated production environments.

Assessing phenotypic diversity of crop genetic resources is essential for crop improvement to identify variation for economic traits to guide cultivar selection and hybridization to develop novel varieties. The present study revealed significant phenotypic differences for grain yield and its components for assayed commercial and mutant wheat genotypes under constrasting moisture environments (Figure 3). Drought stress induced earliness but reduced grain yield in the test genotypes. In addition, yield component traits were also impaired due to drought stress (Table 4). The contrasting responses of the test genotypes under drought and irrigated conditions allowed for the identification of drought-tolerant wheat genotypes for rainfed environments. For example, LMA16, LMA44, and LMA53 were identified as high yielders under drought stress conditions and recorded grain yields of 26.64, 24.01 and 23.40 g/plot (Table 5). These mutant lines outperformed the commercial check varieties, such as SS0166 and SST0117, which are cultivated and adapted to drought-prone wheat-producing areas of South Africa. Further, these genotypes possessed essential yield contributing traits such as shorter plant stature, higher root biomass, and longer spikes (Table 4). These mutant lines are recommended for cultivation in semi-arid areas of South Africa following multi-environment studies against standard commercial varieties. Further, these mutants could be utilized for breeding programs to enhance drought tolerance. On the contrary, mutant lines LMA5 and LMA16 were the high-yielders under irrigated conditions and produced a grain yield of 42.62 g/plot. These are ideal candidate genotypes for cultivation under irrigated conditions. These mutants exhibited 23% higher grain yield compared to the widely cultivated commercial wheat variety SST015, commonly grown under irrigated wheat-producing areas of South Africa. These genotypes possessed essential yield-component traits including productive tillers, root biomass, and thousand-seed weight and are also recommended for multi-environment trials for registration and commercialization. 

Understanding trait correlations is a vital procedure in crop improvement programs to guide simultaneous selection and improvement of desirable traits, and accelerate the development of novel and high-performing varieties. Plant height is an important agronomic trait related to yield performance [61]. In the present study, there were a moderate association between plant height and grain yield, implying limited progress to improve yield gains through selecting for plant height. Productive tiller number is an important indicator that determines wheat productivity and yield potential. A high number of productive tillers is ideal in wheat as it is linked to high biomass production, high spike density, and grain yield [62]. Strong and positive correlations were revealed between productive tillers and grain yield under both drought-stressed and non-stressed conditions (Figure 4) suggesting that selecting for increased tillering capacity could be an effective strategy for improving yield gains. The mutants LMA19 and LMA37 with high tiller numbers are ideal for production under dryland wheat production.

Roots are an essential organ for water and nutrient uptake, which are necessary for crop growth and development. Roots also play a key role in sensing environmental changes and then triggering a response to such stresses [63]. Optimization of root systems is an efficient approach to enhance water and fertilizer use efficiency, supporting crop productivity under adverse conditions [64]. Root biomass showed a high positive correlation with grain yield only under drought stress conditions, suggesting that enhanced root development under drought stress enhances drought tolerance and yield development. Therefore, optimized breeding for high root biomass could enhance wheat productivity in the era of climate change and the mutant lines such as LMA16, LMA6, LMA47, LMA53, LMA2, and LMA42 with high root biomass production under drought stress environments are ideal candidates for future breeding initiatives in South Africa.

Shoot biomass development is an essential indicator to predict yield development. Shoot biomass was highly correlated with grain yield under drought stress conditions, suggesting that increased above-ground biomass favours yield development in the studied wheat population. Therefore, breeding and selection for increased shoot biomass could enhance radiation-use efficiency and boost wheat yields [65]. The mutants LMA19 and LMA5 exhibit higher shoot biomass under drought stress conditions.

Spike length is a key contributor of yield potential in wheat [66]. In the present study, spike length was highly correlated with grain yield under drought stress conditions. This association could be attributed to the potential of high root biomass to extract moisture into deeper layers of the soil under water stressed conditions, thus enhancing spike elongation, florets development and high kernels numbers. Thousand-seed weight is an important wheat grading parameter for quality analysis and directly influences grain yield. In the present study, thousand-seed weight showed moderated and positive correlations with grain yield under drought-stressed and irrigated conditions. Mutant lines such as LMA2, LMA 16 and LMA42 with high root biomass sustained plant hydration and photosynthetic activity during grain development, maintaining continuous assimilate supply to the grains leading to higher thousand seed weight under drought stress. Selecting for seed weight may not lead to significant yield improvements in the studied wheat germplasm. Tanglegram analysis under drought stress and non-stressed conditions revealed one genotype and 5 genotypes that maintained their position, respectively, indicating they maintain stable traits and strong genetic and phenotypic linkages. In contrast, the reduced number of stable genotypes under drought stress indicates that drought stress conditions cause phenotypic plasticity that is not directly linked to SSR marker. This inconsistency indicates the difficulty of genotype-environment interactions during drought and highlights the importance of integrating both molecular and phenotypic data for select stable genotypes [67]. 

## 5. Study Limitations and Future

The selected SSR markers are diagnostic and representative and provide useful genetic differentiation. However, the depth of genetic coverage may be limited compared to genome-wide SNP markers. Therefore, it is necessary to validate findings with high-density SNP markers to increase marker density and resolution. The genotype by environment effects may not have been fully captured because of the few locations; therefore, multi-season trials are necessary for assessing stability and genotype × environment interaction.

## 6. Conclusions

The present study determined genetic diversity, phenotypic diversity, and their interrelationships among selected mutant lines and commercial varieties. High genetic and phenotypic diversity was revealed among check l and mutant wheat varieties. Genotypes LMA16, LMA44, and LMA53 were highly productive under drought stress conditions and are recommended for dry-land wheat production areas of South Africa following multi-environment evaluations. Mutants LMA5 and LMA16 were the best yielders under irrigated conditions and recommended to irrigated wheat production environments in South Africa following multi-environment testing. The identified mutants are genetically unique and differentiated and can serve as valued genetic resources for future wheat improvement programs in South Africa to improve grain yield and drought stress tolerance.

## Figures and Tables

**Table 1:** List of wheat mutant lines and check varieties used in this study.

Entry No.	Genotype	Pedigree	Source
1	LMA2	Mutant	ACCI
2	LMA5	Mutant	ACCI
3	LMA6	Mutant	ACCI
4	LMA8	Mutant	ACCI
5	LMA15	Mutant	ACCI
6	LMA16	Mutant	ACCI
7	LMA19	Mutant	ACCI
8	LMA21	Mutant	ACCI
9	LMA22	Mutant	ACCI
10	LMA29	Mutant	ACCI
11	LMA31	Mutant	ACCI
12	LMA37	Mutant	ACCI
13	LMA42	Mutant	ACCI
14	LM43	ROLF07*2/6/PVN//CAR422/ANA/5/ BOW/CROW//BUC/PVN/3/YR/4/TRAP#1	CIMMYT
15	LMA44	Mutant	ACCI
16	LMA47	Mutant	ACCI
17	LMA53	Mutant	ACCI
18	SS0166	PBR	Sensako
19	SST0117	PBR	Sensako
20	SST015	PBR	Sensako

ACCI—African Centre for Crop improvement; CIMMYT—International Maize and Wheat Improvement Center; PBR—Plant Breeder’s Right. * indicates repeated crossing (backcrossing).

**Table 2:** List of the ten SSR markers used in the study.

Marker Name	Chromosome Location	Primer Sequence
Xgwm132	6A	F: ACCAAATCGAAACACATCAGG R: CATATCAAGGTCTCCTTCCCC
Xgmw484	2D	F: ACATCGCTCTTCACAAACCC R: AGTTCCGGTCATGGCTAGG
XWMC596	7A	F: TCAGCAACAAACATGCTCGG R: CCCGTGTAGGCGGTAGCCTCTT
Wmc179	6A	F: CATGGTGGCCATGAGTGGAGGT R: CATGATCTTGCGTGTGCGTAGG
GWM337	1D	F: CCTCTTCCTCCCTCATTAGC R: TGCTAACTGGCCTTTGCC
Wms169	6A	F: ACCACTGCAGAGAACACATACG R: GTGCTCTGCTCTAAGTGTGGG
Wms30	2D	F: ATCTTAGCATAGAAGGGAGTGGG R: TTCTGCACCCTGGGTGAT
Wmc177	2A	F: AGGGCTCTCTTTAATTCTTGCT R: GGTCTATCGTAATCCACCTGTA
Wmc532	2B	F: GATACATCAAGATCGTGCCAAA R: GGGAGAAATCATTAACGAAGGG
Wmc78	3B	F: AGTAAATCCTCCCTTCGGCTTC R: AGCTTCTTTGCTAGTCCGTTGC

F—Forward, R—Reverse primer.

**Table 3:** Genetic diversity parameters generated by 10 SSR markers.

Markers	Product Size (bp)	Na	Ne	I	Ho	He	F	PIC
WMS30	217–244	2.00	1.93	0.67	0.89	0.48	−0.83	0.49
Xgwm132	97–244	2.00	2.00	0.69	1.00	0.50	−1.00	0.50
Xgwm484	170–192	3.00	2.17	0.71	0.33	0.40	0.31	0.68
XWMC596	159–175	3.00	2.31	0.76	0.11	0.43	0.77	0.83
WMS169	143–157	2.00	1.79	0.61	0.76	0.43	−0.69	0.43
WMC179	216–376	3.00	2.53	0.90	0.98	0.57	−0.76	0.77
GWM337	189–204	1.00	1.27	0.21	0.00	0.15	1.00	0.28
WMC532	178–197	2.00	1.67	0.37	0.00	0.22	1.00	0.50
WMC78	266	1.00	1.00	0.00	0.00	0.00	0.00	0.00
WMC177	200–209	2.00	1.56	0.43	0.16	0.30	0.50	0.61
Mean	-	2.10	1.82	0.54	0.42	0.35	0.24	0.51
SE	-	0.17	0.15	0.08	0.09	0.05	0.15	0.09

Na = number of observed alleles; Ne = number of effective alleles; I = Shannon’s information index; Ho = observed heterozygosity; He = average gene diversity within genotypes; F = fixation index; PIC = polymorphic information content; SE = standard error.

**Table 4:** Mean squares and significant tests for agronomic traits of 20 wheat genotypes evaluated in the glasshouse and field environments under drought-stressed and non-stressed conditions.

Change	d.f.	DTH	DTM	PH	PTN	RB	RSR	SB	SL	TB	TSW	GY
Rep	1	41.00 *	302.50 *	0 ns	3.86 ns	0.97 ns	0.00 ns	442.29 *	0.00 ns	845.3 *	18.97	5.67 ns
Genotype (G)	19	173.50 **	171.80 **	146.83 **	57.77 **	46.96 **	0.00 *	665.76 **	6.75 **	960.89 **	54.19 **	213.76 **
Water regime (W)	1	9.51 ns	10,530.02 **	1163.61 **	868.81	574.77 **	0.01 *	27,490.10 **	0.28 ns	35,594.95 **	2381.70 **	7523.40 **
Environment (E)	1	13,634.56 **	41,473.60 **	50,673.62 **	12,147.32 **	1493.82 **	0.04 **	13,381.70 **	846.98 **	14,525.25 **	9905.34 **	7789.16 **
G × W	19	15.41 *	17.64 ns	23.36 ns	5.93 ns	14.10 ns	0.00 ns	77.31 ns	0.97 ns	145.04 ns	14.27 *	27.70 ns
G × E	19	15.68 *	44.11 ns	180.60 **	48.97 **	22.92 *	0.00 ns	149.51 *	2.43 *	240.85 **	35.83 **	222.45 **
W × E	1	71.5 *6	416.03 **	108.28 *	537.14 **	36.08 ns	0.00 ns	1440.00 **	1.85 ns	2822.40 **	130.52 **	29.63 ns
G × W × E	19	6.39 ns	52.67 *	17.99 ns	4.816	5.85 ns	0.00 ns	77.72 ns	1.25 ns	151.47 *	21.60 *	65.32 *
Residual	79	7.715	29.21	22.05	3.542	11.75	0	75.6	1.061	86.48	8.282	29.99

Rep: replications; df: degrees of freedom; DTH: days to 50% heading; DTM: days to 90% maturity; PH: plant height; PTN: productive tiller number; RB: root biomass; RSR: root–shoot ratio; SB: shoot biomass; SL: spike length; TB: total biomass; TSW: thousand-seed weight; GY: grain yield; *: significant at 5% probability level; **: significant at 1% probability level; ns: non-significant.

**Table 5:** Principal component scores, proportion and cumulative variance of agronomic traits assessed among 20 bread wheat genotypes evaluated under drought-stress (DS) and non-stress (NS) conditions across field and glasshouse environments.

	Drought Stressed		Non-Stressed		
Traits	PC1	PC2	PC1	PC2	PC3
DTH	0.29	**−0.41**	0.25	0.05	**−0.67**
DTM	0.26	**−0.55**	0.29	0.06	**−0.58**
GY	**0.32**	0.22	**0.31**	0.02	0.27
PH	**0.31**	0.29	**0.33**	−0.03	0.02
PTN	**0.30**	0.15	**0.36**	−0.08	0.10
RB	**0.34**	0.21	0.21	**−0.62**	0.04
RSR	0.24	**0.51**	−0.07	**−0.73**	−0.13
SB	**0.33**	−0.21	**0.36**	0.07	0.24
SL	**0.30**	0.02	**0.34**	0.20	−0.02
TB	**0.33**	−0.14	**0.36**	0.09	0.17
TSW	0.29	−0.09	**0.31**	−0.15	0.16
Eigenvalue	7.41	1.18	6.82	1.77	1.00
Proportion of total variance (%)	67.35	10.68	61.97	16.11	9.06
Cumulative variance (%)	67.35	78.03	61.97	78.08	87.14

DTH, days to 50% heading; DTM, days to 90% maturity; PH, plant height; PTN, productive tiller number; SB, shoot biomass; RB, root biomass; TB, total biomass; RSR, root–shoot ratio; SL, spike length; TSW, thousand-seed weight; GY, grain yield; bold-face fonts denote significant loading scores.

## Data Availability

The data supporting the conclusions of this study can be obtained from the corresponding author (J.M.) upon reasonable request.
